# Cross-market spillovers amid policy uncertainty era of China’s economy

**DOI:** 10.1371/journal.pone.0352237

**Published:** 2026-06-26

**Authors:** Yun Song, Kai-Hua Wang, Hong-Wen Liu, Nicoleta-Claudia Moldovan

**Affiliations:** 1 School of International Business, Qingdao Huanghai University, Qingdao, China; 2 School of Economics, Qingdao University, Qingdao China; 3 Department of Humanities & Tourism, Rizhao Polytechnic, Rizhao, China; 4 Department of Finance, Business Information Systems and Modelling, Faculty of Economics and Business Administration, West University of Timisoara, Timisoara, Romania; Kean University, UNITED STATES OF AMERICA

## Abstract

This study meticulously discusses spillover effects among agricultural futures (AGF), crude oil prices (CRO), and the U.S. dollar (USD) during the volatile phase of China’s economic policy (CEPU), utilizing the quantile connectedness approach. The empirical findings show that, in the face of economic instabilities and tail incidents, AGF, CRO, USD, and CEPU exhibit significantly higher return spillover at extreme quantiles in comparison to mean one. Furthermore, the dynamic properties of AGF, CRO, USD, and CEPU are empirically substantiated, and certain crises obviously enhance these spillover effects. The primary innovation involves the integration of CEPU into the analytical framework of the agriculture-energy-financial system. This integration illustrates the interdependencies of the system across a variety of market conditions through dynamic quantile shifts, thereby expanding the theoretical scope. This study recommends actions for different market participants, such as an cross-nation economic cooperation and financial market tracking mechanism.

## Introduction

Amid contemporary global economic landscape, the uncertainty of economic policy (EPU) has evolved into a dominant external shock that drives heightened volatility across global commodity markets [1]. As the world’s primary reserve currency and the main invoicing currency for bulk commodities, the US dollar (USD) acts as a critical transmission channel for cross-market spillovers [2]. Amid escalating geopolitical tensions, widespread trade protectionism and ongoing restructuring of global supply chains, EPU has become a persistent systemic risk that reshapes investor expectations, interacts with USD fluctuations, and transmits shocks to crude oil prices (CRO) and agricultural futures (AGF) through expectation transmission, safe-haven flows and USD-denominated pricing mechanisms [3,4]. Under such combined shocks, the traditional energy-agriculture linkage has shifted from a simple cost-push relationship to significant nonlinear co-movement and multi-layered risk contagion [5], further amplified by the deepening financialization of commodities. As global biggest energy importer and major agricultural consumer, China’s policy adjustments and vulnerability to USD fluctuations, both exert substantial influence on global market stability [6]. Thus, this paper constructs a unified framework of EPU, USD, CRO and AGF to investigate their dynamic connectedness and time-varying spillovers, which enriches the theoretical understanding of cross-market spillover and provides empirical support for safeguarding food security and macroeconomic resilience.

As an emerging economy, China possesses distinctive attributes that render it a compelling research focus. First, China’s economic strategies are characterized by significant ambiguities. EU anti-dumping actions, the trade dispute between China and the US, and other trade protectionist policies have jointly created a challenging external economic environment, compelling frequent adjustments to its economic policies [7,8]. Meanwhile, China’s economic growth has slowed down, accompanied by deep-seated structural imbalances [9], which has spurred innovative reforms to speed up adjustment and promote fundamental optimization, further elevating the uncertainty of economic policies. Second, China faces prominent challenges in the international oil market. Due to limited local oil production and swift economic growth, it has emerged as the world’s biggest net importer of crude oil [10]. It is largely dependent on imports to satisfy its needs; in the first half of 2020, imports of crude oil accounted for almost 73% of total consumption [11]. More seriously, owning to lacking oil pricing power, China passively endures the fluctuations in crude oil prices. Third, China is the biggest importer of agricultural goods worldwide. In 2019, the value of imported agricultural goods surpassed that of the US and the EU at $133.1 billion [12].

This paper analyses the cross-market spillovers among agricultural futures (AGF), crude oil price (CRO), U.S. dollar index (USD) in era of China’s economic policy uncertainty (CEPU), covering period from October 2004 to September 2025 and utilising quantile connectedness approach. The study addresses two core research questions: first, to examine the time-varying spillover connectedness across variables and distinguish between spillover transmitters and receivers; second, to investigate whether such spillover effects exhibit significant differences under normal versus extreme market conditions. This paper further constructs theoretical framework among AGF, CRO, USD and CEPU through cost-push, supply-demand and expectation theories. CEPU transmits variance to AGF via distorted global supply-demand balance and pessimistic market expectations, while CRO transmits variance to AGF through cost transmission and biofuel substitution. CEPU and CRO form a bidirectional feedback loop that amplifies mutual return fluctuation, and the USD acts as a core pricing mediator to inversely regulate commodity demand and prices via exchange rate movements. We provide the following hypotheses: Hypothesis 1: there exist dynamic spillover among AGF, CRO, USD and CEPU; Hypothesis 2: The spillover presents heterogeneity across different quantiles; Hypothesis 3: EPU would exacerbate fluctuations of the AGF; Hypothesis 4: CRO would intensify return fluctuation of the AGF

The major empirical findings indicate that the association between CEPU and other variables is significantly stronger under extreme market conditions than in regular periods. Amid market instability, CEPU correlates significantly with corn and cotton prices, while oil prices are strongly associated with all agricultural futures. In typical markets, oil prices correlate most strongly with Soybean prices, but more closely with Wheat prices in favorable conditions. Variables exhibit time-varying characteristics, with major crisis events amplifying spillover effects; under extreme markets, variables alternate as spillover transmitters and recipients, whereas CEPU acts as a distinct spillover recipient in stable markets.

There are some contributions when comparing to existing literatures. First, this research develops a cohesive theoretical framework based on transmission mechanisms. It integrates cost-push, supply-demand balance, and expectation theories to clarify the interconnections among EPU, CRO, AGF and USD, as no established theory conclusively elucidates these interactions [13,14]. Furthermore, we add to the theoretical framework by analysing time-varying reactions to major external shocks and examining spillover effects under different market circumstances (normal versus exceptional). Second, this study specifically focuses on China and investigates the impact of its EPU on global energy and agricultural markets. The current literature predominantly highlights EPU in industrialized nations [15]. China is naturally more susceptible to changes in world prices since it is the most populated country, the second-largest economy, and a significant importer of energy and agricultural items. Consequently, the CEPU possible affects global commodity prices in distinctive manners [16]. This focus amplifies research on China’s practical experiences and augments theoretical understanding in this domain. Third, this study utilizes the newly developed quantile-VAR spillover methodology to examine cross-market spillovers. It can recognize spillover across various market conditions and several time horizons, surpassing conventional single-time-domain analyses [13,17]. This methodology provides policymakers with a robust toolset for maintaining price stability. Moreover, investors and stakeholders may manage risk exposure and create portfolio strategies to reduce potential future losses by comprehending the mechanisms of return spillovers between markets.

The structure of the paper is as follows. Section 2 looks at the relevant literature. Section 3 introduce theoretical framework, method and data. Section 4 shows empirical findings and related discussion. Section 7 summarize findings and gives policy recommendations.

## Literature review

### CEPU and agricultural market

Against rising global EPU driven by the COVID-19 pandemic and trade protectionism [18,19], China’s agricultural market, characterized by long production cycles and high price volatility, is vulnerable to EPU shocks [20–22]. Key studies and methodologies: Hao and Ki-Seong [21] used a TVP-VAR model, finding time-varying EPU impacts on agricultural prices and heterogeneous product responses. Zhang et al. [23] applied Survival Analysis, showing EPU weakens agricultural import stability. He et al. [24] confirmed EPU distorts agricultural resource allocation (e.g., non-agricultural land transfer), affecting supply and prices. Jiang et al. [25] used percentile correlation and frequency spillover analysis to reveal short-term EPU risk spillovers with product heterogeneity. Gui et al. [26] adopted a TVP-VAR-SV model, noting China’s EPU has stronger impacts on futures volatility than global EPU, amplified by commodity financialization. Song et al. [13] used quantile connectedness, finding enhanced mutual influence among EPU, agricultural futures, and energy prices under high volatility.

### CEPU and energy market, especially oil

The existing literatures widely discuss the impacts of China’s EPU on the oil market, with existing studies identifying positive and negative effects via diverse methodologies. Positively, Wang and Chen [27] integrated EPU into an LSTM-based oil price forecasting framework, validating its predictive value. Lin and Luo [28] used the QARDL model, finding high EPU drives investors to hold crude oil futures for hedging, supporting short-term prices. He and Xu [24] confirmed EPU drives green transformation. Han et al. [29] used the DSGE model to demonstrate that rising China’s EPU stimulates national strategic petroleum reserve replenishment and long-term crude oil demand expansion. Akadırı et al. [30] used the AR(1)-GARCH(1,1) model, combined with wavelet nonparametric quantile causality tests, to reveal the bidirectional causal relationship between economic uncertainty and oil supply as well as economic activity shocks. Negatively, Liu et al. [31] applied the KAN-GARCH-MIDAS model, showing EPU amplifies low-frequency oil market volatility. Sarker [32] used event study and volatility measurement methods, revealing tariff-induced EPU increases oil price volatility.

### CEPU and financial market, especially US dollar

China’s EPU affects the US dollar market via three aspects, verified by distinct methodologies. The first aspect is exchange rate. Liu [33] used the TVP-VAR model, finding China’s EPU causes RMB depreciation (with time-varying characteristics). Wang et al. [34] adopted the Network Connectedness Approach, showing EPU boosts USD safe-haven demand. Yao [35] used Effective Transfer Entropy and the rolling window method, concluding EPU leads to “short-term USD decline and long-term rise”. The second aspect is asset pricing. Lee et al. [36] used ICAPM cross-sectional tests, finding EPU explains 40% of US bond return variation. Ghosh & Hossain [37] used panel regression, identifying short-term negative links between EPU and US stock returns. Guo et al. [38] applied quantile regression, showing EPU increases USD asset holdings and market liquidity. The third aspect is spillovers. Guo et al. [39] used frequency decomposition and the spillover index model, finding EPU causes temporary USD appreciation. Zhao et al. [40] used the quantile time-frequency connectivity model, noting short-term expectation and long-term trade/investment transmission channels.

Most present research predominantly centers on paired relationships, such as EPU and AGF, which lacks a cohesive theoretical framework. Moreover, previous studies overlooked the distinction between extreme conditions and the usual state, neglecting the information contagion impact among the three variables. This research aims to build a theoretical framework for the identified variables and offers theoretical basis for subsequent empirical analysis. Furthermore, we employ quantile connectedness to thoroughly examine cross-market spillover among CEPU, AGF, CRO, and USD across varying situations and timeframes. It can offer directional solutions for stabilizing commodity prices and developing suitable economic policies.

### Theory, methods and data

#### Theoretical analysis.

This section establishes a comprehensive theoretical framework involving EPU, AGF, CRO, and USD, with their complex interaction pathways, with their complex interaction pathways clearly reflecting the intrinsic links between the four variables. First, EPU exerts a significant impact on AGF through two main channels. On the one hand, it distorts the global supply-demand balance of agricultural products, as elevated EPU is directly linked to economic risks, which causes importing nations to build up reserves and agricultural exporting nations to cut exports in order to protect domestic food security, which exacerbates supply constraints and demand growth [5]; on the other hand, it shapes market participants’ psychological expectations, leading to unpredictable behaviors such as delayed investment and consumption that directly trigger AGF market [41]. Second, CRO transmits variance to AGF directly by increasing production and transportation costs. It includes energy-intensive inputs like fertilizers and agricultural machinery, which in turn push up agricultural production costs and drive AGF up [42,43], and indirectly through biofuel substitution, which redirects agricultural land and resources to biofuel production, thereby affecting AGF. Third, EPU and CRO form a mutual interaction. EPU influences CRO by altering economic fundamentals and financial market participants’ behaviors [44], while CRO fluctuations lead to changes in inflation and production costs, prompting policymakers to introduce new economic policies that elevate EPU. Fourth, the US dollar, as the dominant pricing currency for crude oil and agricultural commodities, mediates their price linkages. US dollar appreciation reduces the purchasing power of non-dollar economies, suppressing CRO and AGF demand, while depreciation enhances it [45]. Consequently, we present the subsequent hypotheses:

Hypothesis 1: there exist dynamic spillover among AGF, CRO, USD and CEPU

Hypothesis 2: The spillover presents heterogeneity across different quantiles

Hypothesis 3: EPU would exacerbate fluctuations of the AGF

Hypothesis 4: CRO would intensify return fluctuation of the AGF

## Methods

This paper employs quantile connectedness method, proposed by Ando et al. [46] and Chatziantoniou et al. [47], examine the quantile propagation mechanism of mentioned variables in theoretical analysis. We provide detailed steps in empirical analysis of quantile vector autoregression (QVAR(p)). First, variable selection and preprocess. This paper selects a set of variables, such as agricultural futures, based on theoretical analysis, and apply the first-order difference to each of them. Second, preliminary test for stationarity. This paper employs unit root tests, include ADF, PP and KPSS tests, to examine the stationarity of variables. Finally, we utilize the QVAR connectedness technique to examine variables’ dynamic spillover and reveal heterogeneity across different quantiles.

With the purpose to evaluate connectedness metrics, we initially construct a quantile vector autoregression, QVAR(p), which is shown below:


$$\begin{array}{c} y_{t}=\mu (\tau )+\sum_{j=\text{1}}^{p}\Phi_{j}(\tau )y_{t-j}+u_{t}(\tau ). \end{array}$$ (1)

$y_{t}$and $y_{t-j}$ are k×1 dimensional endogenous variable vectors. τ is between [0, 1] and represents the quantile. p stands for the lag length of the QVAR model. μ(τ) is a k×1 dimensional conditional mean vector. $\Phi_{j}(\tau )$ is a k×k dimensional QVAR coefficient matrix. $\mu_{t}(\tau )$ demonstrates the k×1 dimensional error vector and has a k×k dimensional variance-covariance matrix ∑(τ). In order to convert the QVAR(p) to its QVMA(∞) representation, this paper utilizes Wold’s theorem: $y_{t}=\mu (\tau )+\sum_{j=\text{1}}^{p}\Phi_{t}(\tau )y_{t-j}+\mu_{t}(\tau )=\mu (\tau )+\sum_{i=\text{0}}^{\infty }\Psi_{i}(\tau )u_{t-i.}$

Afterwards, the H-step ahead Generalised Forecast Error Variance Decomposition (GFEVD) of Koop et al. [48] and Pesaran and Shin [49] is calculated, illustrating the shock’s impact from j to i:


$$\begin{array}{c} \overset{g}{\underset{ij}{\Psi }}(H)=\frac{\sum \overset{-\text{1}}{\underset{ii}{\left(\tau \right)}}\sum_{h=\text{0}}^{H-\text{1}}{\overset{\prime }{\underset{i}{(e}}\Psi_{h}(\tau )\sum \left(\tau )e_{j}\right)}^{\text{2}}}{\sum_{h=\text{0}}^{H-\text{1}}\left(\overset{\prime }{\underset{i}{e}}\Psi_{h}(\tau )\sum (\tau )\Psi_{h}{\left(\tau \right)}^{\prime }e_{i}\right)} \end{array}$$ (2)

$$\begin{array}{c} \overset{g}{\underset{ij}{\tilde{\Psi }}}(H)=\frac{\overset{g}{\underset{ij}{\Psi }}(H)}{\sum_{j=\text{1}}^{k}\overset{g}{\underset{ij}{\emptyset }}(\text{H})} \end{array}$$

$e_{i}$depicts a zero vector at position *i*th with unity. The two equalities that result from this normalisation are as follows: $\sum_{j=\text{1}}^{k}\overset{g}{\underset{ij}{\tilde{\Psi }}}(H)=\text{1}$ and $\sum_{i,j=\text{1}}^{k}\overset{g}{\underset{ij}{\tilde{\Psi }}}(H)=k.$

To get the information of the overall impact variable *i* has on all other variables *j*, the total directional connectedness TO others is computed:


$$\begin{array}{c} \overset{g}{\underset{i\to j}{C}}(H)=\sum_{j=\text{1},i\neq j}^{k}\overset{g}{\underset{ji}{\tilde{\Psi }}}(H) \end{array}$$ (3)

Then, the effect of perturbing all other variables *j* on variable *i* is assessed using the total directional connectedness FROM others:


$$\begin{array}{c} \overset{g}{\underset{i\leftarrow j}{C}}(H)=\sum_{j=\text{1},i\neq j}^{k}\overset{g}{\underset{ij}{\tilde{\Psi }}}(H) \end{array}$$ (4)

The net total directional connectedness, which may be thought of as the net effect variable I has on the network under analysis, is the consequence of the differences between the total directional connectedness TO others and the total directional connectivity FROM others.


$$\begin{array}{c} \overset{g}{\underset{i}{C}}(H)=\overset{g}{\underset{i\to j}{C}}(H)-\overset{g}{\underset{i\leftarrow j}{C}}(H) \end{array}$$ (5)

The final connectedness, namely the adjusted total connectedness index (TCI), which falls into [0, 1], and is shown below.


$$\begin{array}{c} TCI(H)=\frac{\sum_{i,j=\text{1},i\neq j}^{k}\overset{g}{\underset{ij}{\tilde{\Psi }}}(H)}{k-\text{1}} \end{array}$$ (6)

Because the degree of network interconnection increases with the TCI, this metric is frequently used as a stand-in for market risk. To sum up, several connection metrics contain quantile VAR model in Equation (1), directional TO (Equation 3), directional FROM (Equation 4), net connectedness index (NET) (Equation 5), and total connectedness index (TCI) (Equation 6).

### Data sources and description

This study covers sample period from January 2005 to September 2025. In 2005, the value of the RMB went up steadily after China set up a controlled floating exchange rate system [50]. Additionally, EPU has also skyrocketed as a result of several China’s events, including the $4 trillion fiscal plan from 2009, the 2015 stock market crash, and the 2018 Trade War [6]. In addition, the data’s time span includes significant global occurrences as the COVID-19 epidemic, trade disputes between the US and China, the European sovereign debt crisis, and the global financial crisis [51,52].

Multiple variables are discussed here. The first variable is China’s economic policy uncertainty (CEPU). The CEPU index was initially developed by Baker et al. [53] and is available at http://www.policyuncertainty.com. It is extensively used in the financial and agricultural markets [14,54]. The second variable is the price of crude oil (CRO). In accordance with Hau et al. [55] and Dai et al. [56], we get the raw data from the Wind database and use the price of Brent crude oil futures to show how volatile real oil prices are. The agricultural commodities futures (AGF), which comprises Soybeans, Corn, Wheat, and Cotton, coming from the Chicago Board of Trade and the New York Futures Exchange. Since numerous studies also use them to estimate agricultural prices, we have chosen them [57,58]. For every variable, the first-order difference of logarithm is calculated because the data must be steady and follow normalcy [59].

Table 1 shows descriptive analysis results. The Cotton had the lowest average value among agricultural commodities. CEPU’s standard deviation was 0.206, which was higher than that of the other variables and suggests that it experiences greater fluctuations than do the other variables. All variables, except Corn and Soybean, exhibit negative skewness showing that the left tail of the distribution is thicker than the right tail. All variables had kurtosis values exceeding 3, indicating that they possess notable leptokurtic properties. The Jarque-Bera test results demonstrated that all variables, with the exception of USD, display a non-normal distribution at a 1% significance level. Table 2 displays the outcomes of the unit root tests: ADF, PP, and KPSS. The findings indicate that all variables exhibit stationarity.

**Table 1:** Descriptive statistics and unit root test.

	Max	Min	Mean	Std. dev	Skewness	Kurtosis	Jarque-Bera
Wheat	0.369	−0.217	0.005	0.075	0.960	6.485	164.274^***^
Cotton	0.284	−0.196	0.002	0.053	0.786	7.931	277.924^***^
Corn	0.122	−0.166	0.004	0.037	−0.027	6.008	93.931^***^
Soybean	0.182	−0.215	0.004	0.057	−0.295	4.831	38.399^***^
WTI	0.722	−0.452	0.007	0.103	0.667	13.691	1204.266^***^
USD	0.062	−0.045	0.001	0.017	0.178	3.519	4.124
CEPU	1.348	−0.504	0.025	0.206	1.769	11.275	840.342^***^

Notes: ^***^ denotes significance at the 1% level.

**Table 2:** Unit root test.

	ADF		PP		KPSS			
	**Statistics**	***p*-value**	**Statistics**	***p*-value**	**Statistics**	**1% CV**	**5% CV**	**10% CV**
Wheat	−13.875(0)^***^	0.000	−13.785(8)^***^	0.000	0.175(5)	0.739	0.463	0.347
Cotton	−10.383(0)^***^	0.000	−10.439(4)^***^	0.000	0.110(7)	0.739	0.463	0.347
Corn	−7.517(2)^***^	0.000	−11.327(1)^***^	0.000	0.344(5)	0.739	0.463	0.347
Soybean	−12.101(0)^***^	0.000	−12.107(1)^***^	0.000	0.184(4)	0.739	0.463	0.347
WTI	−12.350(0)^***^	0.000	−12.013(5)^***^	0.000	0.066(9)	0.739	0.463	0.347
USD	−11.404(0)^***^	0.000	−11.392(4)^***^	0.000	0.041(6)	0.739	0.463	0.347
CEPU	−14.843(1)^***^	0.000	−23.412(5)^***^	0.000	0.223(69)	0.739	0.463	0.347

Notes: ^***^ denotes significance at the 1% level. Numbers in bracket of ADF indicates lag length. Numbers in bracket of PP and KPSS indicates bandwidth.

**Fig 1:**
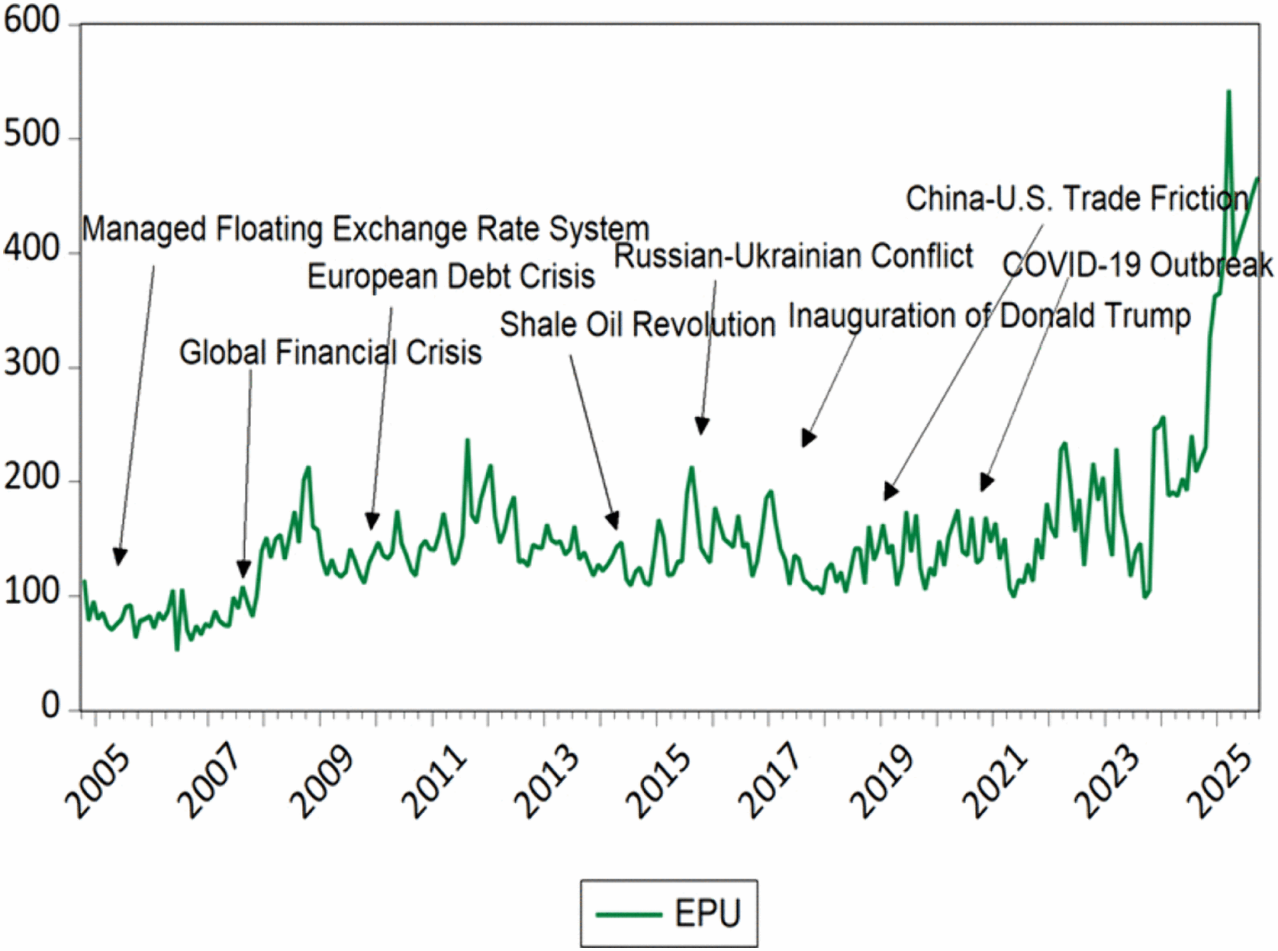
The trend of economic policy uncertainty.

Fig 1 shows the trend of economic policy uncertainty. China introduced a controlled floating exchange-rate system and a share-trading reform in 2005, whereas the United States levied a punitive tariff of 27.5% on its imports, raising EPU [50]. EPU originally increased and subsequently decreased as a result of the 2008 global financial crisis. EPU reached its zenith after Donald Trump’s presidency in January 2017. EPU growth reached a new high in 2018 as a result of trade concerns between the US and China. EPU increased in 2020 as a result of the COVID-19 pandemic [60]. EPU dramatically declined with the onset of the post-pandemic period and the steady impact of China’s preventative and control efforts.

**Fig 2:**
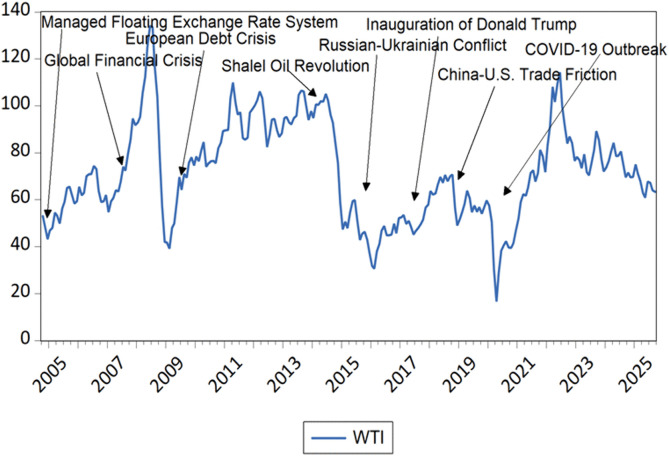
The trend of crude oil price.

Fig 2 shows the trend of crude oil price. The 2008 financial crisis caused a significant fall in the CRO. Events like the shale oil revolution and the Russia-Ukraine war caused the CRO to sharply decline between 2014 and the start of 2016 [4]. At the beginning of 2020, the CRO saw an unprecedented fall as a result of the COVID-19 epidemic and the energy price issue. After April, the world economy rebounded, OPEC tightened its control over oil production, and the price of oil gradually rose as global public health crises began to be handled [61].

**Fig 3:**
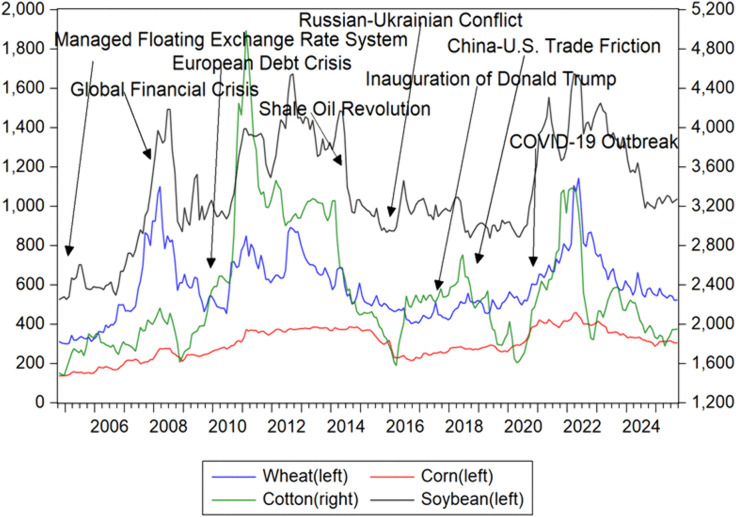
The trend of agricultural futures.

Fig 3 shows the trend of agricultural commodity price. In 2006, extreme weather, rising oil price, and the advancement of biofuels all contributed to a significant spike in AGF. The global financial crisis in 2008 caused the AGF to exhibit a notable decline [62]. The AGF generally increased in 2010 as a result of trade protection measures and extreme weather. The AGF hit its lowest point in recent memory in 2014 because of the global harvest of Soybean, the US dollar’s rising, and China’s economic unpredictable growth [63]. Following May 2020, the AGF steadily rose to a new peak due to the possibility of a decline in global grain production and disruption of the trading chain.

**Fig 4:**
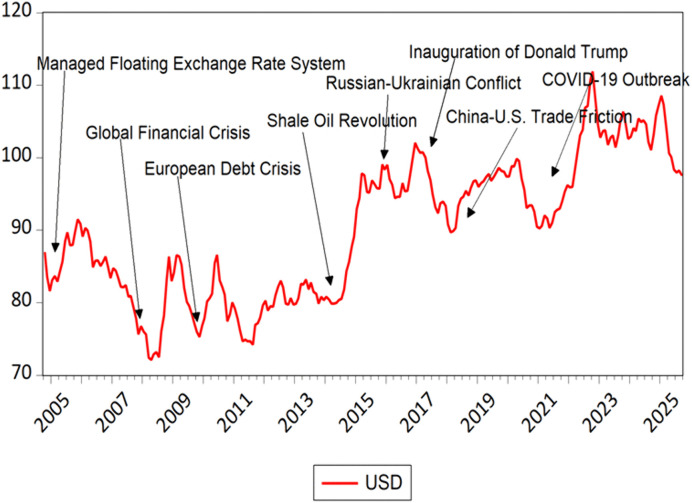
The trend of U.S. dollar.

Fig 4 shows the trend of U.S. dollar index. The financial crisis in 2008 caused the USD to experience significant fluctuations [64]. The European debt crisis in 2010 led to capital flowing from Europe to the United States, which contributed to a rebound in the USD. The Federal Reserve’s loose monetary policy and the Shale Oil Revolution led to an overall upward trend in the USD [65,66]. In 2020, the outbreak of COVID-19 increased global risk aversion and also contributed to an appreciation of the USD [67].

### Empirical analyses

#### Static analysis.

Table 3 presents the return spillovers both to and from all variables at quantiles of 0.1 (lower), 0.5 (median), and 0.9 (higher). The overall connectivity of the median quantile (54.33%) was significantly lower than that of the lower (80.60%) and upper quantiles (83.38%). The outcome lends credence to the notion that the return fluctuation of the extreme quantile (distribution tails) is greater. This finding suggests that under severe circumstances, the spillover connection between CEPU, CRO, AGF, and USD is closer. A number of significant crises have occurred, including global financial crises, European debt crises, Sino-US trade disputes, and COVID-19. The effect of these crises on the economy and commodities markets has only grown [7,50]. For example, the extreme effects of Sino-US trade war deeply affect China’s the macro-economy, industry development, and energy consumption [68]. Besides, market players’ expectations and feelings were impacted by COVID-19, which caused sudden swings on the market for commodities futures [69]. Additionally, commodity costs can offer valuable information for economic policy-making, particularly for fundamental commodities like crude oil and agricultural products [66,70]. China raised financial subsidies, eliminated agricultural tariffs, and expanded the use of developing information technology in reaction to the sharp rise in agricultural prices [71]. In order to deal with the volatility of oil prices, China additionally creates ladder pricing mechanisms, introduces financial subsidies, and optimizes monetary policies by creating a green finance system [72]. To sum up, extreme events frequently induce synchronized market reactions, flight-to-safety behavior, and unanticipated policy interventions, which collectively amplify the transmission of shocks across these variables. During such periods, the tail dependence among CEPU, CRO, AGF, and USD becomes more pronounced than under normal conditions, thereby resulting in heightened spillover connectivity. Consequently, extreme circumstances not only elevate individual market fluctuation but also intensify cross-market linkages.

**Table 3:** Spillovers across different quantiles.

	Wheat	Cotton	Corn	Soybean	WTI	USD	EPU	FROM
Panel A: τ = 0.1								
Wheat	18.08	11.97	14.90	16.70	14.20	12.72	11.43	81.92
Cotton	11.95	18.08	15.29	15.76	14.57	14.15	10.20	81.92
Corn	12.38	13.32	20.60	15.30	14.64	13.57	10.19	79.40
Soybean	13.05	12.47	15.00	19.69	15.27	13.88	10.65	80.31
WTI	11.47	13.03	15.53	15.91	20.81	13.37	9.88	79.19
USD	11.57	12.33	14.90	15.24	15.12	20.03	10.82	79.97
EPU	12.46	12.07	14.41	15.16	14.02	13.40	18.48	81.52
TO	72.89	75.18	90.01	94.06	87.82	80.10	63.16	564.23
NET	−9.03	−6.74	10.62	13.75	8.63	1.13	−18.36	TCI = 80.60
Panel B: τ = 0.5								
Wheat	46.30	8.41	9.21	14.16	7.30	5.74	8.88	53.70
Cotton	9.51	43.00	10.34	10.98	8.39	10.43	7.35	57.00
Corn	8.42	9.75	47.13	10.47	8.85	9.72	5.65	52.87
Soybean	12.64	8.41	10.03	42.31	8.95	9.81	7.84	57.69
WTI	7.48	10.22	9.26	10.73	45.18	10.34	6.78	54.82
USD	7.85	7.59	9.49	9.92	11.44	45.76	7.95	54.24
EPU	10.06	6.57	7.46	9.99	7.80	8.09	50.02	49.98
TO	55.97	50.97	55.80	66.24	52.73	54.14	44.45	380.30
NET	2.28	−6.03	2.94	8.55	−2.09	−0.11	−5.54	TCI = 54.33
Panel C: τ = 0.9								
Wheat	17.38	13.65	13.96	13.51	14.52	13.57	13.40	82.62
Cotton	15.09	16.09	13.76	13.06	14.87	13.48	13.66	83.91
Corn	14.68	14.48	15.70	13.27	14.13	13.71	14.04	84.30
Soybean	15.32	14.22	13.01	15.82	14.19	13.97	13.47	84.18
WTI	14.45	13.99	13.26	13.06	17.45	14.16	13.64	82.55
USD	14.91	13.62	13.39	12.51	14.32	16.91	14.34	83.09
EPU	14.39	13.66	12.73	12.73	15.56	13.95	16.99	83.01
TO	88.83	83.63	80.10	78.14	87.59	82.84	82.55	583.67
NET	6.21	−0.28	−4.21	−6.04	5.04	−0.25	−0.46	TCI = 83.38

Notes: The TCI value denotes the total spillover index, and the higher the value, the stronger the connectedness between the variables; The NET value denotes the net directional connectedness. If the value is positive, it means that the variable is a net risk transmitter. On the contrary, it acts as a net risk receiver.

Additionally, we discovered an intriguing feature: when we switched between the quantiles, for certain variables, the net overflow’s sign altered. To characterize the variable identities, we employ transmitters and receivers. The quantity of data received by the variable or communicates to the marketplaces of the other variables determines their economic relevance. For instance, the Wheat accepted shocks in the 0.5 quantile and delivered information in the 0.1 and 0.9 quantiles. The Cotton communicated data in the lower quantile after receiving it in the upper and median quantiles. These findings illustrate the asymmetrical relationship of return among EPU, the CRO, and the AGF. All conditional quantiles showed consistent trends in the net return spillovers for the CRO, EPU, and the other two agricultural commodities (corn and soybeans). Across all quantiles, the EPU and the CRO had a net influx of data. Price changes in the market for agriculture (Soybean, Corn, Wheat, and Cotton) have an impact on EPU and the CRO, particularly in a very negative market. Commodity prices are a useful tool for formulating economic policy and can offer new information regarding the status of the economy [73]. For instance, rising oil prices immediately increase living and production costs, causing fundamental changes inside an economy that compel the administration to modify its economic strategies [74]. Thus, changes in geoponic commodity prices will send economic data to EPU. Furthermore, we observed that Soybean and Corn were information transmitters at each of the three quantiles, but EPU was a net information receiver. China is the largest importer and consumer of soybeans worldwide. In 2021, its reliance on imports exceeded 80% [75]. China developed various new economic policies in response to the Soybean fluctuation in order to maintain stability the market for geoponic commodities. Thus, it is possible to explain The Soybean’s risk effects on China’s EPU. Furthermore, the Cotton received information in the middle quantile with great strength. As a result, during normal times, the Cotton is a greater issue for stakeholders and policymakers.

**Fig 5:**
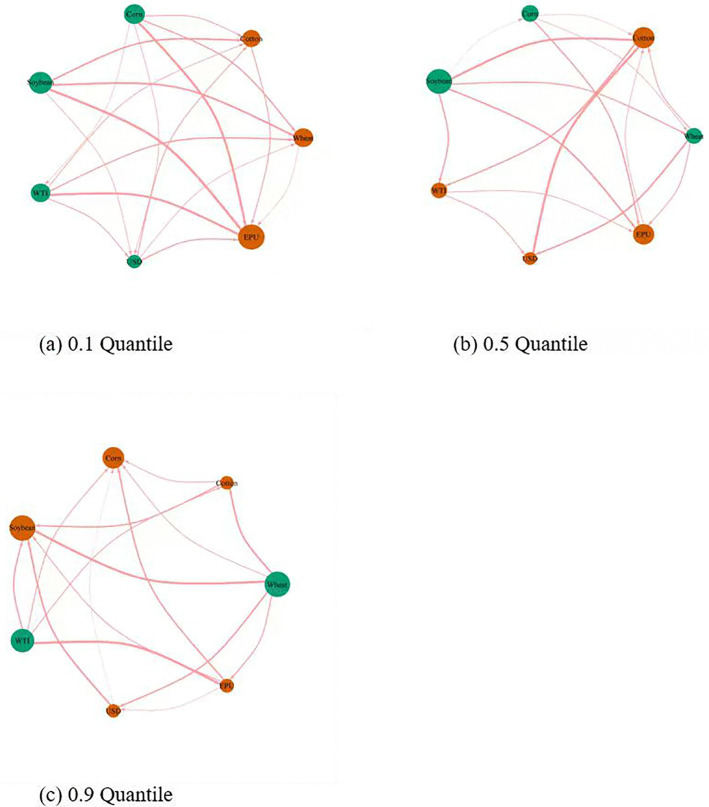
Full-sample connectedness networks. Note: Arrow directions denote the spillover transmission direction, and arrow thickness reflects the spillover scale.

The pairwise network-directional connectedness between each variable is represented separately in the network diagram in Fig 5. Compared to the median quantile, the net connectivity was substantially larger in the high and low quantiles. In contrast to the 0.5 quantile, the spillover lines in the high and low quantiles were thicker. For example, the interconnectedness of EPU, CRO, and AGF was darker in color in the analogous network lines at the 0.1 and 0.9 quantiles. This suggests that in extremely dire circumstances, the three markets’ spillover links were stronger. As a result, from the perspective of network connectedness, the relationship between the variables in the quantiles was significantly different. In the 0.5 quantile, we found that EPU had little connection with other variables; however, in the 0.1 and 0.9 quantiles, the connected variables (CRO and AGF) showed a significant increase, leading to a greater association. This finding indicates that EPU played a more significant role in informing the markets for agricultural commodities and crude oil in harsh conditions. The CRO return has a major effect on China’s EPU because it is the global biggest oil importer [76]. China creates laws to lessen the detrimental consequences of systemic risks and latent contagion effects in response to CRO fluctuation. Additionally, China’s EPU had a significant impact on global markets during the 2008 financial crisis, which may explain the larger EPU spillover effects with the other two elements in severe marketplaces [77]. Moreover, the primary objective of macroeconomic policy is price stabilization. Variations in AGF will directly affect prices for commodities and production costs since agricultural goods are necessary inputs in the manufacturing of many commodities. Therefore, in reaction to shifts in agricultural commodity prices, the government will adjust economic policies, supporting the AGF’s spillover effect on EPU.

However, a major weakness in the static study is the assumption that the interconnectivity of EPU, CRO, and AGF will be steady across the sample duration. Changes in the relationships between events in politics and financial market volatility are disregarded by this presumption. It is difficult to utilize fixed parameter models for the entire sample duration since commodity futures markets are dynamic [78]. We examine the time-varying connectivity measures in order to resolve this issue.

### Dynamic connectedness

**Fig 6:**
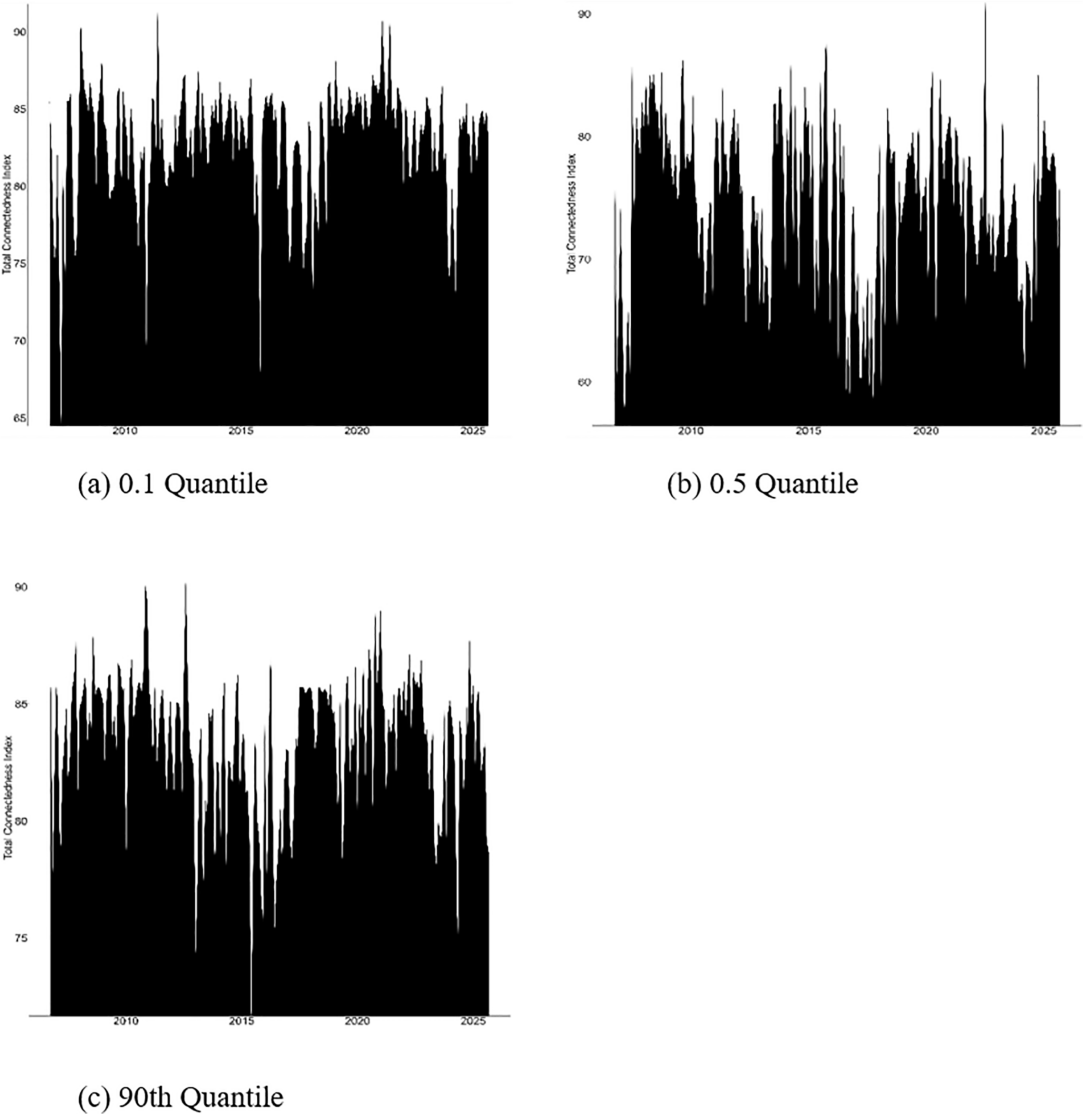
Dynamic rolling window connectedness. Note: The shaded area means spillover. The higher the position of the curve on the area of the shaded part, the higher spillover index at a point in time.

In this part, we use Quantile-VAR to conduct a rolling analysis to determine temporal spillover fluctuations under various conditional probabilities. The spillover network system’s total return spillover across variables is shown by the TCI. The system is more connected when the TCI score is higher. Fig 6 demonstrate the temporal changes in the TCI at the 0.1, 0.5, and 0.9 quantiles.

Compared to the median quantile (0.5), the low (0.1) and high (0.9) quantiles had significantly higher connection values, the data clearly indicate that the TCI is time-varying. The median quantile’s TCI fluctuated quite a little, from 35% to 65%. On the other hand, the high quantiles (from 65% to 85%) and low quantiles (from 65% to 83%) varied considerably less over time. These findings demonstrate that the variables in the return distribution’s various quantiles have asymmetric and time-varying return spillovers. The spillovers peaked between 2008 and 2012, when they reached 80%. The price spillover effect between several product types was reinforced by trade and financial deregulation [79]. Because of severe weather events like Hurricane Katrina in the U.S., as well as catastrophic floods in India that lower the output of staple crops like corn and rice, the agricultural market experiences broad price increases. In addition, several countries have imposed trade limitations to limit agricultural exports and encourage imports. The Argentine government increased the export duties on wheat and soybeans from 20% to 28% and 27.5% to 35%, respectively, in November 2007. The international economy was additionally impacted by the 2008 global financial crisis and the 2011–2012 Eurozone crisis, which significantly increased the spillover impacts of the AGF, CRO, and EPU. Compared to the high TCI observed during severe shocks, the global economy rebounded between 2013 and 2014, and the TCI overall declined [80]. The return spillover was continuously high prior to 2016, primarily because of EPU, which could be caused by a number of variables. For example, the collapse of China’s financial market in August 2015, political strikes and unrest in November 2016, growing anxiety over the shadow banking industry, and growing worries about a recession in China’s economy in 2012. Between 2017 and 2019, there was a notable decline in the TCI across all quantiles. This result explains the more obvious benefits of diversifying the market for agricultural commodities during times of crisis, such as the Organization of Petroleum Exporting Countries (OPEC) price reduction agreement and the ongoing antagonistic relationship between China and the U.S. Dynamic spillovers started to rise dramatically when COVID-19 started in early 2020, especially in harsh markets.

### Net total and pairwise spillover

**Fig 7:**
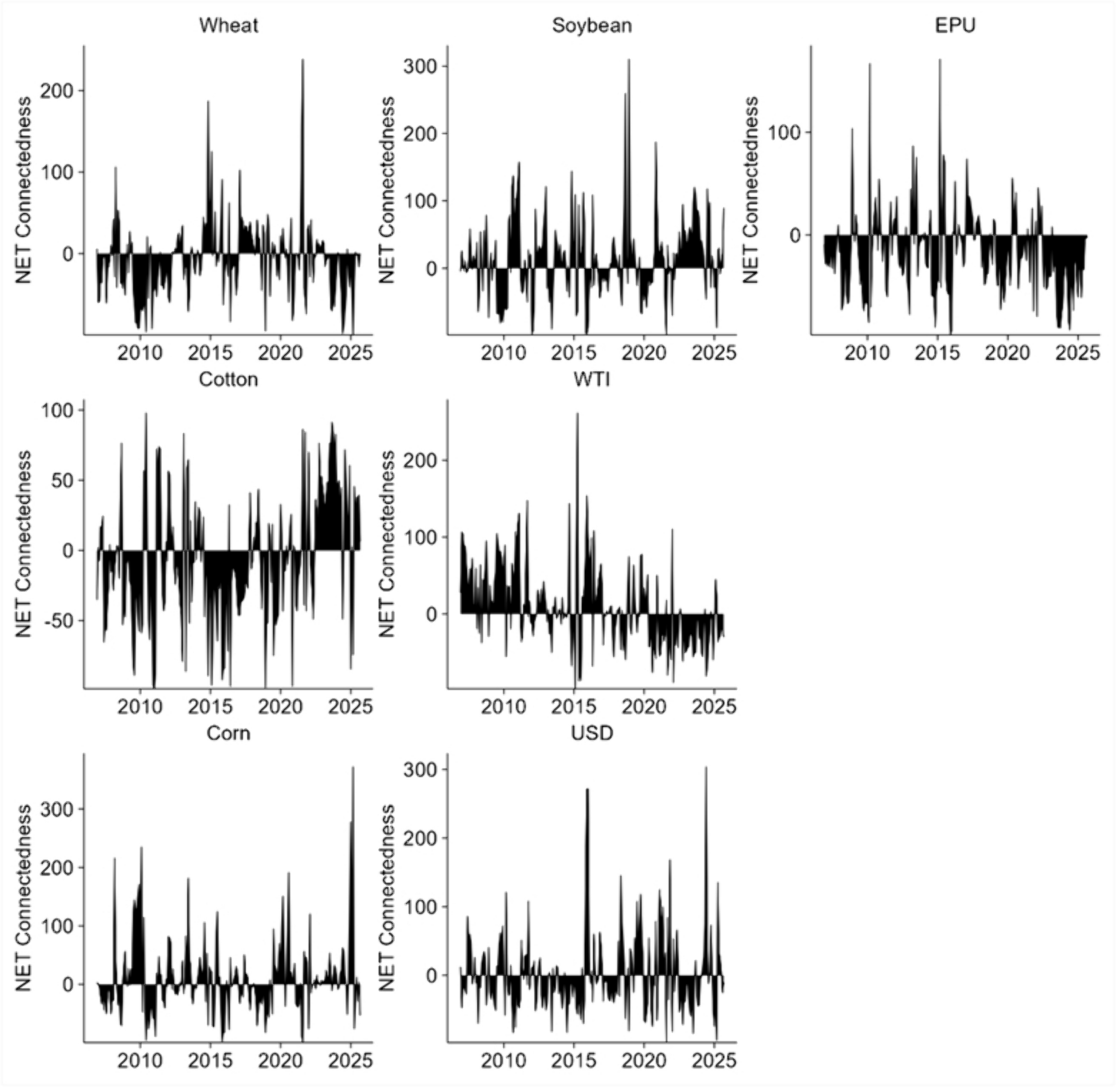
The net spillover at 0.1 quantile. Note: The shaded area means spillover. The higher the position of the curve on the area of the shaded part (both positive and negative), the higher spillover at a point in time.

**Fig 8:**
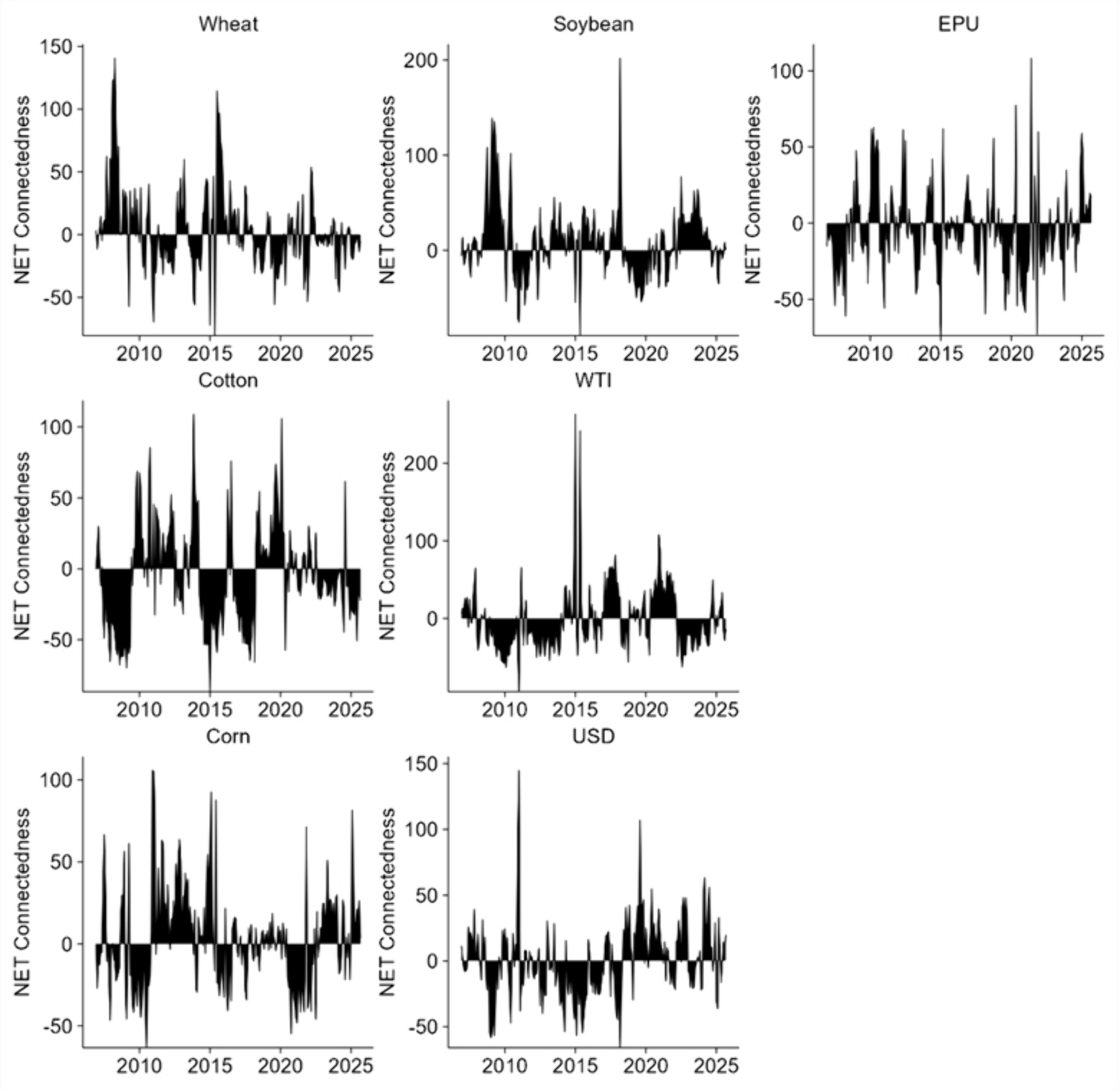
The net spillover at 0.5 quantile. Note: The shaded area means spillover. The higher the position of the curve on the area of the shaded part (both positive and negative), the higher spillover at a point in time.

**Fig 9:**
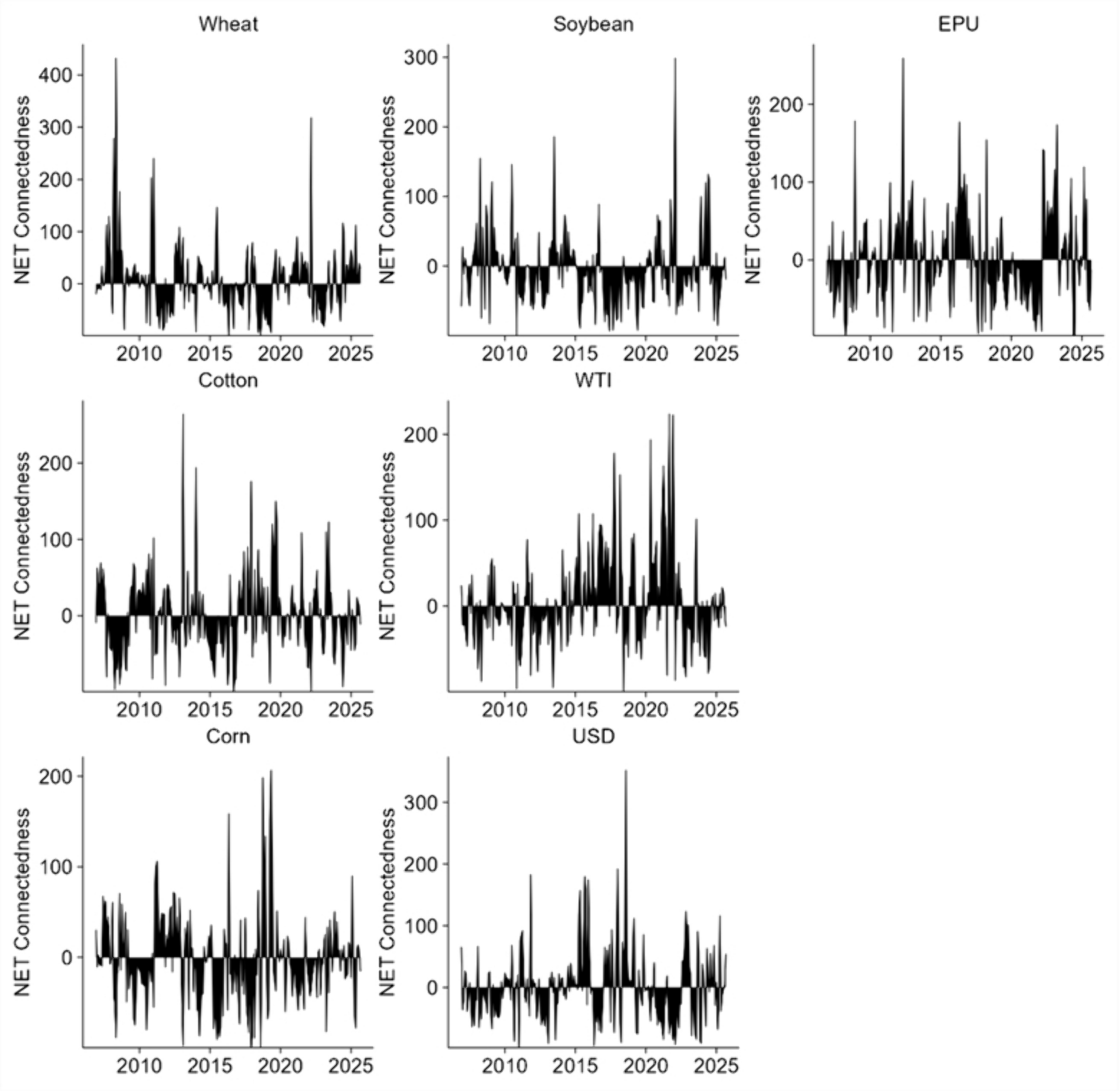
The net spillover at 0.9 quantile. Note: The shaded area means spillover. The higher the position of the curve on the area of the shaded part (both positive and negative), the higher spillover at a point in time.

The relationship between rolling net return and connectedness at three different quantiles of 0.1, 0.5 and 0.9 are shown in Figs 7–9. This approach makes it easier to classify variables into systemic risk, net transmitters, and net recipients of information. This section’s dynamic method enables the identification of likely transitions between the two variables, as opposed to the classification discussed in above section. They may ultimately serve as the system’s net transmitters and net receivers. The net transfer effects for the receiver are shown by positive values, whereas negative values imply the opposite. The Corn was an evident transmitter and the EPU was a clear receiver during ordinary financial conditions, as shown in Fig 8. For the duration of the time, the remaining variables were not significant. However, for unexcepted incidents, the patterns of net return spillovers were very different, with more net return spillovers than those that occurred under normal markets. Between 2007 and 2011, EPU was a clear beneficiary of risk and knowledge under extremely unfavorable circumstances, as illustrated in Fig 7. Some important events, such as the bankruptcy of Lehman Brothers in 2008, the new round of quantitative easing policy in the U.S. and the European debt crisis, may produce shocks on China [81]. To combat these shocks, China launched some economic policies, including the 2009 budget plan worth $4 trillion. Furthermore, in 2015, EPU demonstrated a notably large spillover impact. This spillover could have been caused by exchange rate reform and the unanticipated devaluation of the RMB in August 2015. Furthermore, between roughly 2008 and 2018, the CRO was a clear source of information and hazards. The 2008 subprime mortgage crisis led to price fluctuations and foam in the oil market. The CRO fell to about $40 per barrel during the crisis after reaching a record peak of over $140 per barrel prior to it [82]. Additionally, OPEC production cuts and geopolitical developments caused oil prices to fluctuate throughout 2018, explaining the increase in CRO spillovers during these two periods. EPU was nearly invariably the information recipient in a good economic circumstance, which is shown Fig 9. Considering that 21% of China’s food output was imported in 2022, the country’s economic growth is susceptible to restrictions on the supply and demand of commodities [83]. China’s agricultural imports have grown from about $10 billion in 2001 to more $120 billion in 2018 after joining the World Trade Organization (WTO), making up over 8% of the world’s food trade [84]. Due to this circumstance, EPU accepts the spillover effect of bulk goods, in this case agricultural commodities and petroleum [85].

These findings offer fresh perspectives on how shocks caused by severe market fluctuations spread. The figures show how the variable’s net return spillover fluctuate over time. Furthermore, we demonstrate that the connection spillovers resulting from large-scale positive/negative shocks cannot be accurately represented by spillover networks evaluated under conditional averages. The spillover effects of different markets worsen during crises, highlighting the necessity of prudential control and monitoring systems for the AGF, CRO, and EPU. Investors and regulators will therefore overlook the significance of fluctuation in severe situations if historical trends centered on average network connectivity persist. As a result, investors and regulators should keep a close eye on the dynamic spillover effects brought on by excessive EPU, CRO, and AGF variations.

**Fig 10:**
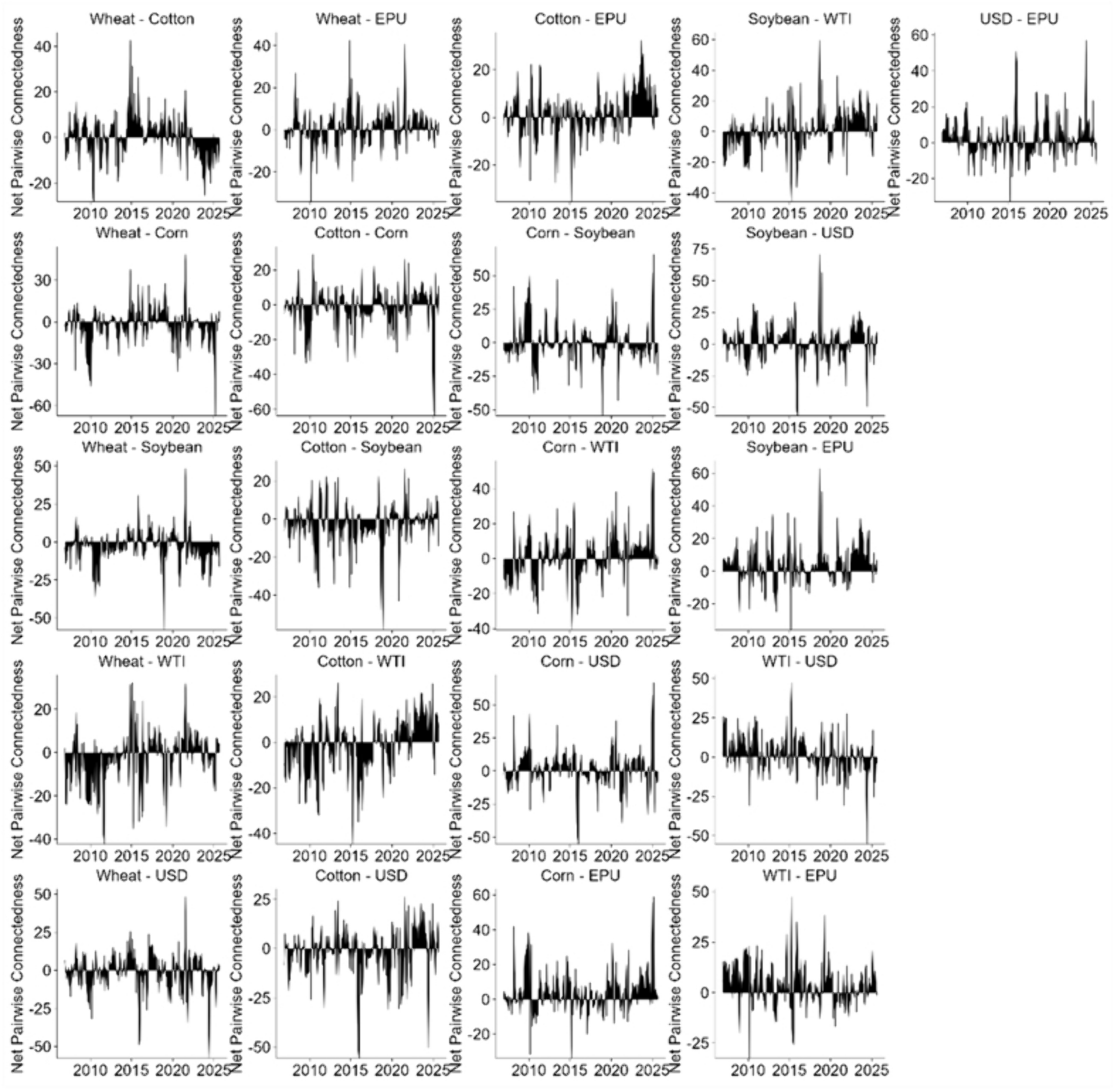
The time-varying net pairwise spillover at 0.1 quantile. Note: The shaded area means spillover. For Wheat-EPU, the shadow area of the figure is below the horizontal coordinate, which means EPU is a receiver of spillover of Wheat. The higher the position of the curve on the area of the shaded part (both positive and negative), the higher spillover between the two variables at a point in time.

**Fig 11:**
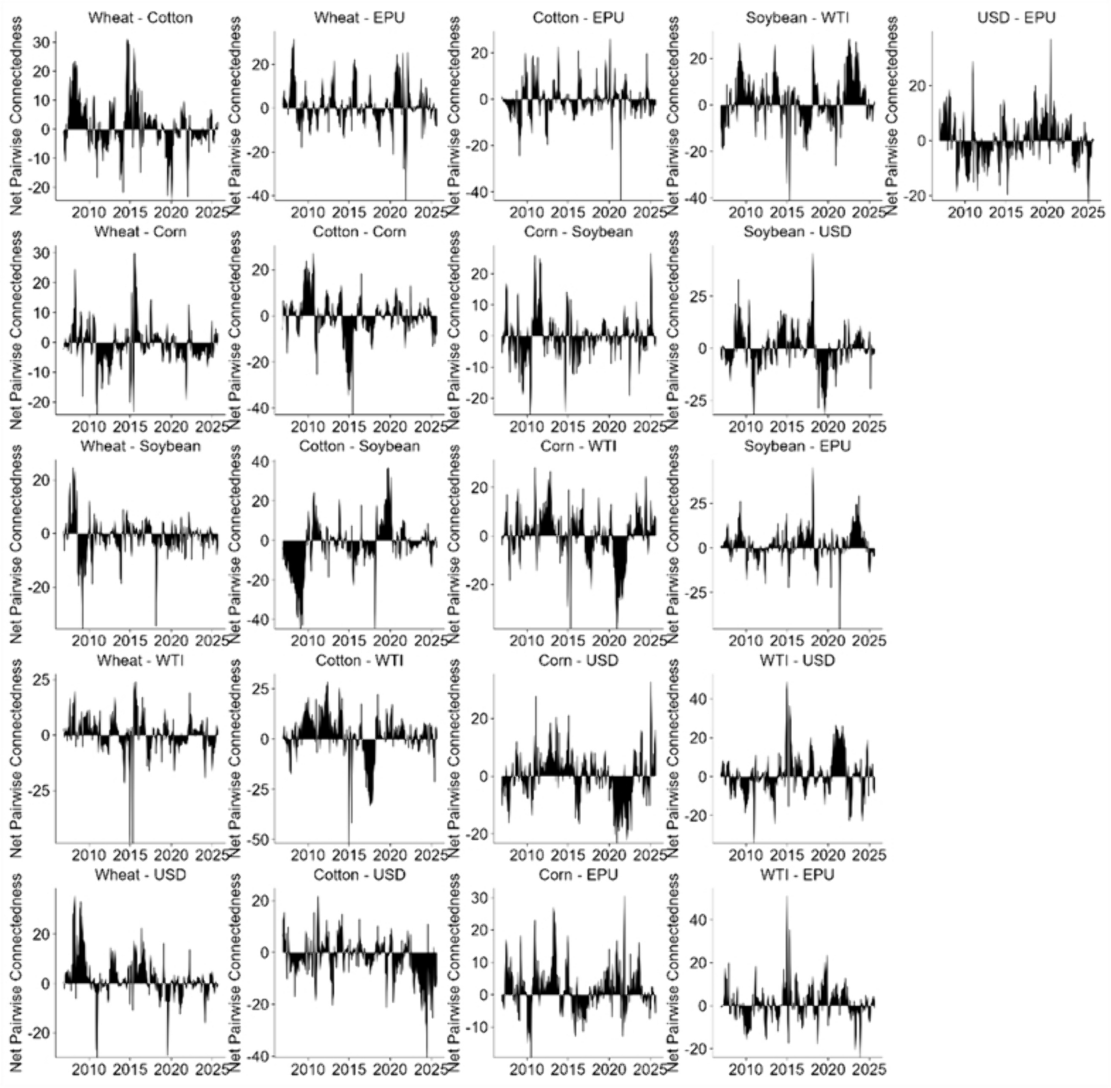
The time-varying net pairwise spillover at 0.5 quantile. Note: The shaded area means spillover. For Wheat-EPU, the shadow area of the figure is below the horizontal coordinate, which means EPU is a receiver of spillover of Wheat. The higher the position of the curve on the area of the shaded part (both positive and negative), the higher spillover between the two variables at a point in time.

**Fig 12:**
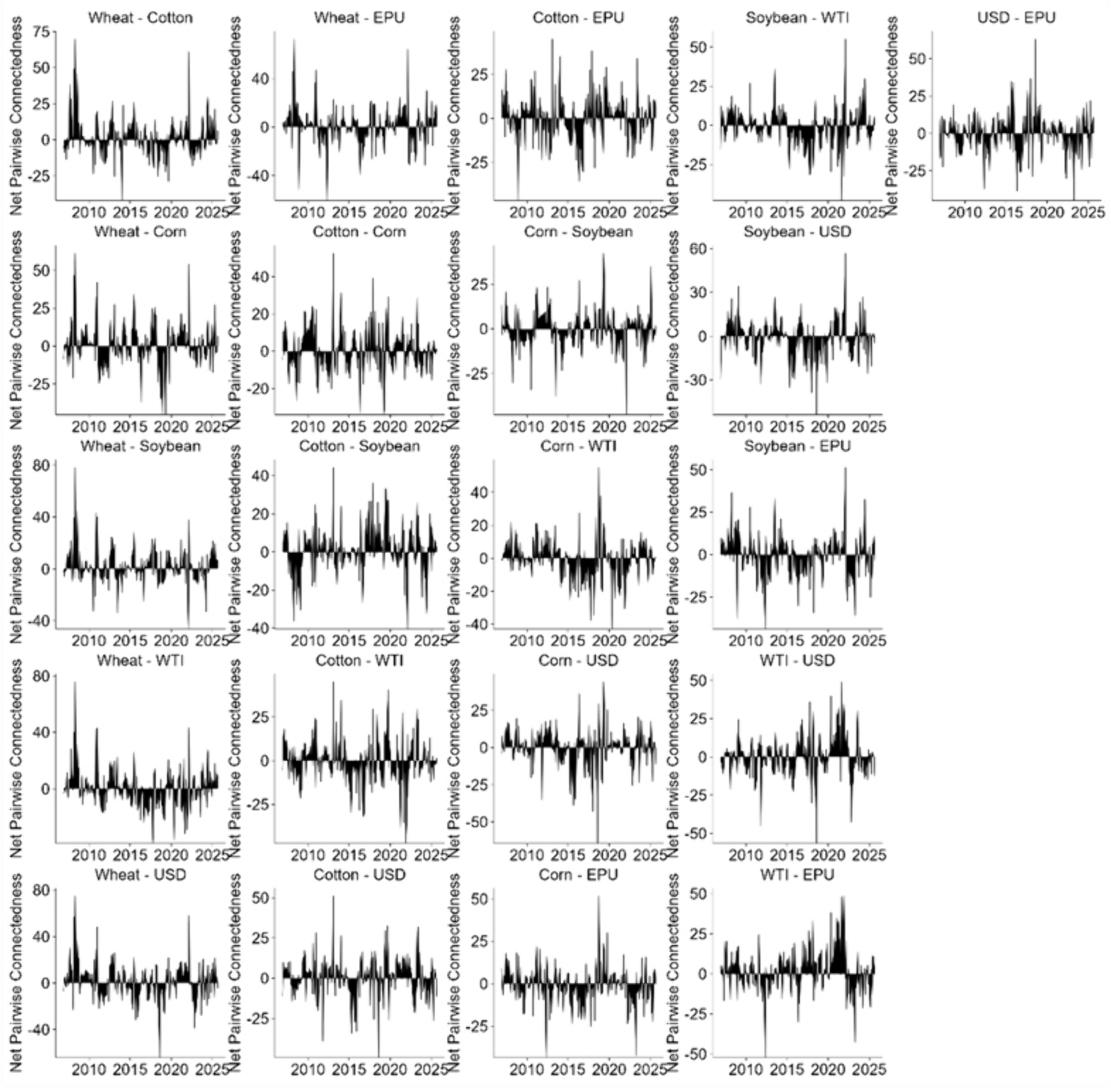
The time-varying net pairwise spillover at 0.9 quantile. Note: The shaded area means spillover. For Wheat-EPU, the shadow area of the figure is below the horizontal coordinate, which means EPU is a receiver of spillover of Wheat. The higher the position of the curve on the area of the shaded part (both positive and negative), the higher spillover between the two variables at a point in time.

We further explore the pairwise connectivity within the network, as presented in Figs 10–12. To determine the significance of EPU and CRO in the agricultural commodity pricing network, we investigate their spillover effects. There was a spillover impact from the AGF and the CRO, as seen in Figs 10 and 11. Because of the drop in oil prices during the COVID-19 epidemic, production decreased. The prices of agricultural products used in the manufacture of biofuel, such corn and soybeans, may rise as a result of this circumstance [86]. The observed rise in agricultural futures might be linked to a surge in biofuel consumption. As one illustrative example, global biofuel output saw a nearly twelvefold expansion over this period, climbing from 228,000 barrels per day back in 1986–2,771,000 barrels per day by 2019 [43]. Simultaneously, several nations are launching biofuel programs to lessen reliance on fossil fuels, mitigate energy and ecological challenges, and transform agricultural products [87]. The CRO and AGF are strongly correlated when biofuels are used in place of oil [88]. Furthermore, the manufacturing and logistics costs of agricultural goods are influenced by the CRO, which in turn transmits variance to AGF [89]. Following 2020, the negative and normal situations’ spillover impacts exhibited opposing trends. Thus, there is a strong correlation between the CRO and AGF, although other factors influence the direction of the overflow. Our primary emphasis is on harsh conditions’ unpredictability. Fig 12 shows that CEPU is net receiver against Soybean in many periods, but the Corn had a smaller impact. Due to China’s extremely low level of Soybean self-sufficiency (below 25%), the country’s domestic Soybean prices are heavily exposed to price fluctuations and market dynamics in importing nations [90]. Consequently, China modified its economic policies in response to shifts in the Soybean, which helps explain why Soybean exerts a stronger influence on EPU. In contrast, China’s Wheat self-sufficiency rate surpasses 95%, making it less vulnerable to external hazards and less likely to generate EPU swings, which is consistent with our empirical findings.

### Robustness test

(1) Altering core variables

We substitute the key variables of oil price and economic policy uncertainty in order to verify the validity of our findings. This study uses the IMF oil price as an alternative oil price metric in accordance with Elekdag et al. [91] and Ghoshray and Pundit [92], who point out that the average of Brent, WTI, and Dubai can reflect the general trend of global energy costs. Furthermore, using People’s Daily and Guangming Daily as data sources, we adopt a new EPU index that was created by Davis et al. [93]. The revised QVAR framework is shown in Model (1), and Table 4 shows the related results. The validity of our findings is confirmed by these updated results, which show that the NET of both EPU and CRO stays negative and increases in size at extreme quantiles, consistent with the baseline values.

**Table 4:** Robustness test.

Variable	Wheat	Cotton	Corn	Soybean	WTI	USD	EPU	TCI
Panel A τ = 0.1								
Model (1)	−3.37	−4.35	2.66	3.11	2.92	8.56	−9.53	83.39
Model (2)	−3.91	−2.09	8.60	15.93	7.08	13.56	−7.31	84.47
Model (3)	−7.29	−7.04	4.26	8.28	0.26	8.76	−7.23	81.76
Panel A τ = 0.5								
Model (1)	8.44	−2.11	2.22	3.01	−2.95	−1.78	−6.83	69.71
Model (2)	7.75	−4.80	5.65	7.87	−6.25	−4.28	−5.94	62.42
Model (3)	6.25	−1.68	2.32	5.51	−5.54	−1.18	−5.68	65.68
Panel A τ = 0.9								
Model (1)	5.93	−1.58	−3.47	−2.10	5.74	−2.74	−1.78	84.16
Model (2)	8.47	−0.85	−2.14	−7.75	7.41	−1.02	−4.12	84.87
Model (3)	3.49	−2.53	−1.88	−2.67	7.55	−2.60	−1.36	83.57

Notes: The TCI value denotes the total spillover index, and the higher the value, the stronger the connectedness between the variables.

(2) Replacing the lag period

To verify the reliability and validity of the empirical results, this paper conducts a sensitivity analysis by adjusting model lag specification to examine whether the core findings are sensitive to alternative lag orders. Following the research framework of Das et al. [94], we re-calculate the model by setting the lag order to 2. The estimating results are shown in Model (2) of Table 4. It is evident that the net directional connectedness and spillover characteristics under the revised lag setting are highly consistent with those derived from the baseline model. This consistency further verifies that the empirical conclusions are not driven by arbitrary lag selection, thereby confirming the strong robustness and credibility of our core findings.

(3) Changing forecast horizon and rolling window size

This study, building on Wang et al. [95], does a sensitivity analysis to determine if the empirical results are affected by varying rolling window sizes and prediction horizons, therefore enhancing the robustness of our conclusions. In the baseline specification, the rolling window and forecast horizon are established at 100 and 10, respectively. To ensure robustness, we re-estimate the model utilising expanded parameters, especially a rolling window of 20 and a prediction horizon of 12. The latest QVAR model, referred to as Model (3), is displayed in Table 4. The results indicate that the behaviour and size of the NET index mostly align with those obtained from the original configuration, so affirming the validity of our conclusions.

### Theoretical and practical implications

Theoretical significance: This study advances the extant scholarly discourse by challenging the static interdependence assumptions prevalent in early studies [96], and delineating critical inferences that deepen theoretical understanding of the interactive mechanisms among CRO, AGF, USD, and CEPU, alongside their multifaceted implications for heterogeneous stakeholders. Significantly, it dissects the non-stationary nature of the CRO-AGF correlation, challenging static paradigms implicitly adopted by studies, such as Fowowe [97], who assumed time-invariant correlations between energy and agricultural markets. Furthermore, the research unveils time-varying tripartite dynamics among these variables, particularly during crisis episodes characterized by intensified risk contagion. This finding not only complements extant literature on cross-market spillover effects that have largely focused on bivariate settings [98], but also furnishes a theoretical cornerstone for cross-market framework for risk governance. Collectively, these contributions extend analytical frontiers beyond static or pairwise dependency structures [99, 78] in the context of energy-agricultural-currency-policy nexuses under extreme events, informing sophisticated risk modeling methodologies and systemic risk governance frameworks.

Practical significance: This study offers evidence-informed, context-specific recommendations to guide strategic decision-making for policymakers, investors, and institutional actors, in contrast to prior studies that often provided generalized, one-size-fits-all policy implications [100]. For governments as regulatory architects, findings underscore the imperative for targeted interventions that account for EPU’s absorptive capacity, a dimension largely overlooked in conventional policy frameworks [101], namely optimizing import diversification, incentivizing technological innovation, and conducting stress-tested policy simulations to safeguard macroeconomic resilience. Individual investors are empowered to refine asset allocation through sophisticated modeling of commodity market interdependencies, with a focus on mitigating spillovers during financial turbulence [102]. Institutional and corporate entities, conversely, must adopt adaptive hedging protocols that supersede static hedging strategies documented in earlier corporate risk management literature [103] and establish multi-layered early warning systems as opposed to single-threshold alert mechanisms [104], capable of tracking time-varying correlation metrics to preemptively navigate systemic shocks and catastrophic risk exposures.

### Conclusion and policy implications

This paper applies a quantile connectivity technique to examine spillover among CEPU, CRO, USD and AGF, trying to capture their interrelationships and time-varying dynamics. According to the empirical findings, overall spillovers might rise significantly in extreme situations. Corn and Wheat primarily act as net spillover originators in the lower quantiles, whereas CEPU and CRO are characterized as net receivers of shocks at both tails of the distribution. The spillover dynamics across variables are influenced by differing economic fundamentals. The strongest interconnections occur during stormy times among EPU, CRO, and Corn, where CRO correlates highly with all farm commodity prices. By contrast, CRO and Soybean matter in normal markets, whereas CRO and Wheat dominate during booms. CEPU serves as the information recipient in a declining market, as indicated by the time-varying study. The lower quantiles demonstrate the most instability in net spillovers relative to the upper and intermediate quantiles. This study innovatively constructed a theoretical framework, revealing spillovers and contagion via a shock-adjusted model in crises, focusing on vulnerable developing nations. It synthesizes cross-sector data to offer actionable risk strategies, transcending traditional analysis through dynamic correlations and quantile insights.

This paper offers specific recommendations for multiple market participants. First, government should deploy stabilization instruments triggered by conditional Value-at-Risk (CVaR) thresholds to mitigate extreme price fluctuations in agricultural commodity markets. When price fluctuation exceeds predefined limits, automatic temporary reserve releases or option-based subsidy mechanisms should be activated. A tiered buffer stock policy for essential agricultural products, specifically grains and edible oils, should be implemented, with minimum and maximum inventory requirements jointly managed by central and local governments. Furthermore, an inter-agency data-sharing platform involving agricultural, energy, and financial regulators should be established to regularly publish cross-market position and price linkage analyses. Second, investors should utilize fluctuation index-based instruments (e.g., OVX, EVX) and cross-asset risk parity strategies to hedge against risk spillovers among energy, financial and agricultural markets. Asset managers shall allocate a predefined minimum share of commodity-focused portfolios to these instruments when rolling cross-market correlation exceeds a specified threshold. When crude oil fluctuation remains elevated for a sustained period, agricultural-exposed products shall increase margin requirements by a fixed percentage and submit stress test reports within a short regulatory deadline. Cross-market circuit breakers should also be introduced to temporarily halt trading in affected agricultural contracts following simultaneous sharp declines in both markets. Third, global organizations need to devise sound energy strategies to ensure crude oil prices stay within appropriate bounds. China should propose a clearly defined price band for Brent crude to OPEC + , within which participating members commit to maintaining prices through coordinated production adjustments. When prices fall below the band’s lower bound for a sustained period, members shall implement a jointly agreed production reduction of a specified minimum volume. The IEA should establish standardized release rules for strategic reserves when prices exceed the upper bound. China should also sign bilateral data-sharing agreements with major oil-producing nations and conduct annual joint emergency response drills.

Owing to restrictions in data accessibility and authorization, the evaluation of agricultural futures, obtaining from the Chicago Board of Trade and the New York Futures Exchange, may be subject to bias, particularly with respect to estimating China’s actual market impact. With China’s commodities market continues to expand, the availability of mature, high-quality data will become increasingly necessary. Regarding methodology, the quantile VAR connectedness approach assumes parameter stability across market conditions. However, during extreme incidents, including financial crises or geopolitical conflicts, parameters may exhibit structural breaks or time-varying characteristics. Future research could extend the model by allowing for time-varying parameters or regime-switching mechanisms to better capture dynamic risk spillovers between agricultural and oil markets.
